# Knowledge, Attitude, and Practice of Skin Care Among Elderly Patients in Riyadh, Saudi Arabia

**DOI:** 10.7759/cureus.43921

**Published:** 2023-08-22

**Authors:** Ghada Bin Saif, Omar A Alsheikh, Nawaf Alkhudhayri, Sulaiman F Alzomia, Sara M Alabdulkareem, Tareq A Alalwan, Joud A Aljebreen, Abdulaziz M Alabdulkareem

**Affiliations:** 1 Dermatology, College of Medicine, King Saud University, Riyadh, SAU; 2 Anesthesiology, King Faisal Specialist Hospital and Research Centre, Riyadh, SAU; 3 Dermatoloy, College of Medicine, King Saud University, Riyadh, SAU

**Keywords:** health, dermatology, geriatrics, elderly, skin care

## Abstract

As people age, the likelihood that they will experience maladies of the skin increases. It is therefore important that older individuals possess the necessary knowledge and proper attitudes and practices regarding their skincare. The purpose of this study was to examine such knowledge, attitudes, and practices among older members of the Saudi Arabian population. The results of the study showed that among the majority of the participants, skincare practices could be considered insufficient and, surprisingly, elderly men undertake better skincare practices than women of this age category. It is recommended that more should be done to educate those within the older Saudi population regarding skin care practices.

## Introduction

Skincare encompasses various practices that help maintain skin integrity, improve its appearance, and alleviate skin conditions. These practices include but are not limited to, nutrition, avoidance of excessive sun exposure, and appropriate use of emollients. In certain situations, such as skin that is either too dry or too wet, dermatitis prevention, and skin injury prevention, skincare should be a regular everyday practice. Skincare is a part of wound healing treatment, radiation therapy, and some medications [1].

Aging is a normal part of life, and the skin degenerates structurally and functionally as people age [2]. The vasculature of the skin deteriorates with age, and because collagen and elastin fibers become scattered and disordered, the supporting dermis also deteriorates. These changes make the elderly more vulnerable to vascular disorders, such as stasis dermatitis, skin injuries, such as pressure ulcers and skin tears, and a declining capacity to repair skin [3]. As a result, skin treatment in late adulthood must be prioritized by clinicians and caregivers [2].

The cumulative effects of intrinsic and extrinsic skin aging increasingly compromise the barrier function of aging skin, resulting in an increase in diseases and disorders over time [4]. Most people over 65 have at least one skin condition, and many have two or more [5]. Sun exposure and ultraviolet radiation are the most significant factors in skin problems. Sun protection is considered one of the most effective primary preventive methods for skin cancer and other skin problems [4].

A study in China found xerosis to be the most common skin problem in the elderly, which can lead to pruritis, asteatotic eczema, and secondary infections. The researchers also discovered that elderly patients often had difficulty applying topical emollients or medications independently. Some of the nurses in the elderly home paid particular attention to assisting the residents with applying moisturizers, significantly reducing the severity of the problems [6].

Other studies conducted in Iran suggest that skin dryness was the most common problem. Furthermore, their expertise, methods, and practices related to skin health could have been better, indicating the need for an accurate and systematic training plan to improve these people's knowledge and practices [7].

The majority of research about the skin within the elderly population focuses on sunscreen use [8], the formation of ulcers in intensive care units [9], skin nursing diagnosis [10], ultraviolet and skin cancer [11], skin modifications versus photoaging [12], sunscreens [13], structural and functional changes of normal aging skin [14], and the study of skin infections [15]. Therefore, this study aims to assess the Saudi Arabian elderly population’s knowledge about, attitude toward, and practice of skin care.

## Materials and methods

A quantitative cross-sectional survey study was carried out to obtain information regarding Saudi elderly patients’ knowledge about, attitude toward, and practice of skin care. This study also aimed to determine the factors that might impact these patients’ level of knowledge, attitude, and practice of skin care.

Participants were selected on the basis of the following inclusion criteria: attendance within geriatric, diabetes, dermatology, or primary care clinics during 2021 and 2022 at King Khalid University Hospital, Riyadh, Saudi Arabia, and an age of 60 years or older. The questionnaire was sent to 1,000 potential participants; 433 patients aged 60 to 89 agreed to participate, providing a response rate of approximately 43%. One hundred ninety-one (44.1%) of the participants were patients from the diabetes clinic, 141 (32.6%) were from the dermatology clinic, 60 (13.9%) were from the geriatric clinic, and 34 (7.9%) were from the primary care clinic. Seven (1.8%) of the participants did not disclose this information. Participants filled out the questionnaire by themselves.

The questionnaire used in this study was an adapted version of the one used by Morowatisharifabad et al. [7]. After these researchers had permission to use their questionnaire, it was first forward translated from Persian into English and then into Arabic. Some of the items on the questionnaire allowed the participants to choose more than one answer.

Questionnaire data were automatically entered into an Excel sheet (Microsoft Corporation, Redmond, WA) and were analyzed using SPSS version 28 (IBM Corp. Armonk, NY). Descriptive statistics regarding demographics, knowledge, attitude, and practice were obtained. Mean practice scores were calculated and compared between age groups (older 60-74, elderly 75-89), gender (male, female), and the highest educational level attained (no formal education, primary school, middle school, secondary school, university) using the t-test and analysis of variance (ANOVA). A Pearson correlation coefficient (r) analysis was also conducted to determine whether a relationship existed between mean attitude and practice scores.

Ethical clearance was provided by the Institutional Review Board of the College of Medicine at King Saud University: IRB approval for Research Project No. E-21-6074.

## Results

The mean age of the participants was 66.82 (SD 4.94). Descriptive statistics related to the participants’ age grouping, gender, education level, marital status, and employment status are reported in Table 1.

**Table 1:** Demographic Characteristics of Participants

		N	%
Age	60-74 (older)	397	91.7
	75-89 (elderly)	36	8.3
Gender	Male	310	71.6
	Female	123	28.4
Highest level of education attained	No formal education	20	4.6
	Primary school	97	22.4
	Middle school	150	34.6
	Secondary school	99	22.9
	University	67	15.5
Marital status	Married	369	85.2
	Unmarried	64	14.8
Employment status	Employed	340	78.5
	Unemployed	93	21.5

Participants were also queried about certain health-related behaviors (smoking, sleep) and extant medical conditions (disease, stress level, allergies, medications). Descriptive statistics related to these questions are also reported in Table 2.

**Table 2:** Information Regarding Participants’ Health-Related Behavior and Medical Conditions

		N	%
Do you have a skin disease?	Yes	302	69.7
	No	131	30.3
Do you smoke cigarettes or other tobacco products?	Yes	137	31.6
	No	296	68.4
How do you score your stress level?	Low	70	16.2
	Medium	287	66.3
	High	76	17.6
On average, how much sleep do you get daily?	Less than 6 hours	44	10.2
	6-8 hours	248	57.3
	8-10 hours	129	29.8
	More than 10 hours	12	2.8
Are you known to have allergies?	Yes	262	60.5
	No	171	39.5
Do you have a disease other than a skin disease?	Yes	332	76.7
	No	101	23.3
Do you take any medications?	Yes	351	81.1
	No	82	18.9

Participants responded to several questions that focused on their level of knowledge about factors and materials that affect skin health, practices that assist in protecting their skin, and symptoms that indicate diseases related to the skin.

Regarding factors and materials that affect skin health, a majority of the participants believed that the sun’s rays are beneficial to the health of the skin (52.2%), stress does not affect skin sensitivity (55.2%), toxins and pesticides are not harmful to the skin (58.2%), using a detergent without gloves is not harmful (61.4%), the sun’s rays are at the greatest intensity in the afternoon (80.8%), tension and nervousness have the most significant impact on skin health among other potentially contributing factors (55.9%), eating spicy food is the eating habit that most negatively affects skin health (56.1%), and primary exposure to cold is the major factor contributing to dry skin (58.7%). To protect the skin, more participants suggested wearing a hat (26.8%), wearing gloves (34.9%), and wearing glasses (43.6%) is more effective in protecting against the sun than sunscreen (24.0%). Concerning symptoms related to diseases of the skin, the majority of participants believed that thinning of the skin is the most representative symptom of skin allergies (52.7%), discoloration is the skin change that best represents a precursor to cancer (53.8%), and thyroid disease is the disease that most affects the health of the skin (50.3%).

Participants were asked questions to determine their attitude toward skincare. Each of the 12 attitude-related questions was posed to elicit an agree or disagree answer, with the additional neutral option indicating the lack of a stance.

The mean attitude scores that were calculated separately for all participants and participants in the older and elderly groups, where disagree, don’t know, and agree were scored as 1, 2, and 3, respectively, on a Likert scale, as seen in Table 3.

**Table 3:** Mean Attitude Toward Skincare Scores M=Mean; SD=Standard deviation

	All participants		Older participants		Elderly participants	
	M	SD	M	SD	M	SD
Attitude	1.88	0.28	1.92	0.30	2.04	0.29

The effect of age group on the attitude of all participants toward skincare was examined using an independent sample t-test. No statistically significant difference was found in attitude for age group as seen in Table 4.

**Table 4:** Effect of Age Group on Attitude Toward Skincare A p-value of less than 0.05 is considered significant. M=Mean; SD=Standard deviation; df=Degree of freedom

	Older		Elderly				
	M	SD	M	SD	t value	df	p
Attitude	1.94	0.30	1.94	0.29	0.15	431	0.88

The effect of gender on the attitude toward skincare of all participants, older participants, and elderly participants was examined using an independent sample t-test and these results are shown in Table 5. No statistically significant difference was found in attitude for gender for any of these groups.

**Table 5:** Effect of Gender on Attitude Toward Skincare A p-value of less than 0.05 is considered significant. M=Mean; SD=Standard deviation; df=Degree of freedom

	Women		Men				
Attitude	M	SD	M	SD	t value	df	p
	All participants						
	1.99	0.31	1.93	0.29	-1.88	431	0.06
	Older participants						
	1.98	0.31	1.93	0.29	-1.73	395	0.09
	Elderly participants						
	2.03	0.36	1.92	0.28	-0.85	34	0.41

The effect of educational level on the attitude toward skincare of all participants, older participants, and elderly participants was examined using a one-way ANOVA, and these results are shown in Table 6. For all participants together and for older participants, no statistically significant effect of educational level on attitude was found. However, for elderly adult participants, a one-way analysis of variance showed that there was a statistically significant effect of educational level on attitude with primary and middle school-educated participants scoring higher than those with higher levels of education. Descriptive data from participants within the illiterate (n = 2) and university (n = 1) groups were not displayed due to their very small sample sizes.

**Table 6:** Effect of Educational Level on Attitude Toward Skincare A p-value of less than 0.05 is considered significant. M=Mean; SD=Standard deviation; df=Degree of freedom

	Primary school		Middle school		Secondary school				
Attitude	M	SD	M	SD	M	SD	F ratio	df	p
	All participants								
	1.88	0.27	1.86	0.32	1.88	0.49	1.75	4,427	0.14
	Older participants								
	1.88	0.28	1.85	0.32	1.89	0.39	1.44	4,391	0.22
	Elderly participants								
	1.92	0.17	1.95	0.33	1.65	0.22	2.84	4,31	0.04

Participants were asked questions to determine their skincare practice. Each of the 14 practice-related questions was posed to elicit an answer on a five-point Likert scale (1= never, 2 = scarcely, 3 = sometimes, 4 = most of the time, 5 = always). Descriptive statistics related to the participants’ answers to these practice questions are shown in Table 7.

**Table 7:** Frequency Distribution of Responses to Practice Questions

	Never		Scarcely		Sometimes		Most of the time		Always	
	N	%	N	%	N	%	N	%	N	%
I use glasses	64	14.8	63	14.6	156	36.1	116	26.9	33	7.6
I use sunscreen	83	19.2	52	12.0	137	31.6	130	30.0	31	7.2
I wear a brimmed hat when I sit down	93	21.5	55	12.7	155	35.8	101	23.3	29	6.7
I use moisturizing creams	76	17.6	47	10.9	163	37.6	93	21.5	54	12.5
I do my work at night so that I am less exposed to the sun	79	18.2	48	11.1	141	32.6	118	27.3	47	10.9
I wear clothes that cover more parts of the body in the sun	64	14.8	73	16.9	151	34.9	108	24.9	37	8.5
I eat fresh vegetables and fruits in my daily diet	69	15.9	63	14.5	120	27.7	136	31.4	45	10.4
I drink 6 to 8 glasses of water every day	69	15.9	58	13.4	198	45.7	75	17.3	33	7.6
I do 150 minutes of exercise and physical activity per week	66	15.2	78	18.0	150	34.6	98	22.6	41	9.5
I visit a doctor if I see a skin problem	68	15.7	86	19.9	163	37.6	89	20.6	27	6.2
In winter, I cover my skin with a hat, gloves, and a mask	79	18.2	84	19.4	135	31.2	91	21.0	44	10.2
In winter, I use sunscreen and moisturizer	60	13.9	105	24.2	141	32.6	94	21.7	33	7.6
I spend some time of the day relaxing and relaxing exercises	56	12.9	77	17.8	157	36.3	116	26.8	27	6.2
When using pesticides on the farm, I use protective equipment such as gloves, masks, and full clothing	57	13.2	61	14.1	138	31.9	129	29.8	48	11.1

The mean practice scores that were calculated separately for all participants and participants in the older and elderly groups were never, scarcely, sometimes, most of the time, and always scored as 1, 2, 3, 4, and 5, respectively, on a Likert scale. as shown in Table 8.

**Table 8:** Mean Skincare Practice Scores M=Mean; SD=Standard deviation

	All participants		Older participants		Elderly participants	
	M	SD	M	SD	M	SD
Practice	2.94	0.97	2.93	0.96	3.08	1.01

The effect of age group on skincare practice was examined using an independent sample t-test. No statistically significant difference was found in attitude for age group as seen in Table 9.

**Table 9:** Effect of Age Group on Skincare Practice A p-value of less than 0.05 is considered significant. M=Mean; SD=Standard deviation; df=Degree of freedom

	Older		Elderly				
	M	SD	M	SD	t value	df	p
Practice	2.93	0.96	3.08	1.01	-0.89	431	0.38

The effect of gender on the skincare practice of all participants, older participants, and elderly participants was examined using an independent sample t-test, and these results are shown in Table 10. For all participants, a statistically significant difference in practice was found for gender with men scoring higher than women. For older adult participants, no statistically significant difference in practice was found for gender. For elderly adult participants, a statistically significant difference in practice was found for gender with men scoring higher than women.

**Table 10:** Effect of Gender on Skincare Practice A p-value of less than 0.05 is considered significant. M=Mean; SD=Standard deviation; df=Degree of freedom

	Women		Men				
Practice	M	SD	M	SD	t value	df	p
	All participants						
	2.77	1.00	3.01	0.95	2.34	431	0.02
	Older participants						
	2.79	1.00	2.98	0.95	1.82	395	0.07
	Elderly participants						
	2.30	0.98	3.23	0.96	2.18	34	0.04

The effect of educational level on the skincare practice of all participants, older participants, and elderly participants was examined using a one-way ANOVA, and these results are displayed in Table 11. This analysis showed no statistically significant effect of educational level on practice for any of these groups. Descriptive data from participants within the illiterate (n = 2) and university (n = 1) groups were not displayed due to their very small sample sizes.

**Table 11:** Effect of Educational Level on Skincare Practice A p-value of less than 0.05 is considered significant. M=Mean; SD=Standard deviation; df=Degree of freedom

	Primary school		Middle school		Secondary school				
Practice	M	SD	M	SD	M	SD	F ratio	df	p
	All participants								
	2.94	0.91	3.06	0.91	2.73	0.96	1.68	4,428	0.15
	Older participants								
	2.92	0.88	3.07	0.89	2.70	0.96	2.12	4,392	0.08
	Elderly participants								
	3.05	1.12	2.96	1.11	3.49	0.36	0.47	4,31	0.76

Attitude scores show a very weak positive correlation with practice scores, r(431) = .154, p = 0.001.

## Discussion

The purpose of this study was to examine the 60+ years old Saudi Arabian population’s knowledge about, attitude toward, and practice of skin care. A majority of these participants demonstrated a perspective that could be considered contrary to that which medical practitioners would hold. For example, many believe that stress does not impact the sensitivity of the skin. However, while the exact mechanism is not fully understood, it has been shown that psychological stress can increase the degree to which skin ages [16]. Results showed that more than half the participants believed in the benefits that can be attributed to exposure to sunlight. Harm from excessive sunlight can come in the form of skin cancer, photoaging, rosacea, and chronic discoid lupus erythematosus [17]. Of particular concern is the finding that almost 60% of the participants did not consider the exposure of the skin to toxins or pesticides to be problematic. Studies have shown the adverse effects on skin health that can result from exposure to 2,4-Dichlorophenoxyacetic acid and paraquat [18] or organochlorine pesticides such as dichlorodiphenyltrichloroethane (DDT) [19]. These adverse effects can include the development of contact dermatitis, urticaria, erythema multiforme, ashy dermatosis, chloracne, hypopigmentation of the skin, and skin cancer [20]. Surprisingly, 56% of the participants believed that eating spicy foods has a negative effect on skin health. Capsaicin, which enters the body through the eating of chili peppers, can serve to mitigate cancer, including cancer of the skin [21]. In line with extant research regarding protective measures against the harmful effects of the sun [22], more participants believed that wearing a hat, gloves, and glasses is better than wearing sunscreen. Another important finding from the study is that the participants believed that discoloration is the primary indication of skin cancer when, in fact, all the options presented in the survey, except for pain, should be considered as potential symptoms for angiosarcoma and nodular melanoma [23,24].

The mean attitude toward skincare score for all the participants and for the older and elderly groups separately leaned more toward disagreement with the survey statements of healthy skin care than with agreement. A similar finding was made with Iranian participants aged 60-99 and 18-55 [7,25]. For elderly adult participants, a one-way ANOVA showed that there was a statistically significant effect of educational level on attitude with primary and middle school-educated participants scoring higher than those with higher levels of education.

The mean skincare practice score tended toward the middle of the Likert scale, which represented a tendency to sometimes practice skincare. Interestingly, the findings showed that the male participants in this study practiced skincare more frequently than the female participants. However, this difference existed only within the elderly population of participants.

Finally, the results of the study showed a weak positive correlation between the attitude toward and practice of skin care. This result mirrors that found in research conducted with older Iranian adults and American collegiate athletes [7,26]. Such a correlation makes sense given the premise that individuals who believe that proper skincare is a path toward better health will endeavor to undertake practices that will avail them of a healthier life.

Medical and cosmetic skincare are the two forms of skin care. Ingredients that can penetrate or change the working of the skin are not used in cosmetics sold over the counter without a prescription. If these ingredients are present, the cosmetic would be labeled a pharmaceutical and could only be sold with a doctor's prescription. However, some ingredients can penetrate the skin but only have a minor impact on the skin. These ingredients, which fall between cosmetics and pharmaceuticals, are called "cosmeceuticals." As a result, the primary distinction between over-the-counter cosmetics and medical skincare products is that the latter can only be prescribed by physicians and are only available by prescription because they contain pharmaceutical-strength ingredients that can change skin functioning [27].

In older adults, preventive skin care involves all practices that include cleaning and caring for the skin to improve health and minimize the risk of developing skin disorders or diseases. Preventive skin care in older adults can be classified as primary, secondary, and tertiary prevention [28,29].

Because the elderly have trouble applying topical emollients, they are less likely to adhere to a treatment plan. This group's socio-physiologic features, such as their physical abilities and whether they have caregivers, should be considered. If they live alone, for example, elderly patients with reduced physical function may need routine foot care or assistance with topical therapies or dressings. Additionally, increased skin fragility, the prevalence of polypharmacy, and the probability that treatments will be required for an extended period should raise safety concerns regarding any drug prescribed. For these reasons, therapeutic regimens commonly used in younger patients may need to be adjusted for the elderly [3].

A limitation of this study is the reliance on self-report data. The collection of data from a survey, by itself, can lead to biases due to the difficulty that people might have in recalling their practices and based on their need to achieve social desirability with respect to their answers about attitude.

## Conclusions

Skincare encompasses various practices. As one ages, so does their skin. Healthy lifestyle choices and good skin care can help improve the quality of life. This study explored the knowledge, attitudes, and practices of members of the Saudi Arabian older and elderly population regarding skincare. The results show that much needs to be done to provide more education for individuals within these population groups that should help to improve their attitudes toward skincare and to encourage them to institute practices that will offer them opportunities for healthier lives.
